# Bayesian dynamic borrowing in group-sequential design for medical device studies

**DOI:** 10.1186/s12874-025-02520-6

**Published:** 2025-03-20

**Authors:** Maria Vittoria Chiaruttini, Giulia Lorenzoni, Dario Gregori

**Affiliations:** Unit of Biostatistics, Epidemiology and Public Health, Department of Cardiac, Thoracic and Vascular Sciences and Public Health, University of Padova, Via Loredan, 18, 35131 Padova, Italy

**Keywords:** Historical information, Bayesian dynamic borrowing, Group sequential design, Noninferiority, Medical device, Self-Adapting mixture prior, Congruence, Incongruence

## Abstract

**Background:**

The integration of historical data into ongoing clinical trials through Bayesian Dynamic Borrowing offers significant advantages, including reduced sample size, trial duration, and associated costs. However, challenges such as ensuring exchangeability between historical and current data and mitigating Type I error inflation remain critical. This study proposes a Bayesian group-sequential design incorporating a Self-Adaptive Mixture (SAM) prior framework to address these challenges in medical device trials.

**Methods:**

The SAM prior combines informative priors derived from historical data with weakly informative priors, dynamically adjusting the weight of historical information based on congruence with current trial data. The design includes interim analyses, with Bayesian decision rules leveraging futility and efficacy boundaries derived using the frequentist spending functions. Effective Sample Size calculations informed adjustments to sample size and allocation ratios between experimental and control arms at each interim. The methodology was evaluated using a motivating example from a cardiovascular device trial with a noninferiority hypothesis.

**Results:**

Four historical studies with substantial heterogeneity were incorporated. The SAM prior showed improved adaptation to prior-data conflicts compared to static methods, maintaining Type I error and Power at their nominal levels. In the motivating trial, the MAP prior was approximated as a mixture of beta distributions, facilitating congruence testing and posterior inference. Simulation studies confirmed the proposed design’s efficiency under both congruent and incongruent scenarios.

**Conclusions:**

The proposed Bayesian Group-Sequential Design with SAM prior offers a robust, adaptive framework for medical device trials, balancing statistical rigor with clinical interpretability. This approach enhances decision-making and supports timely, cost-effective evaluations, particularly in dynamic contexts like medical device development.

**Supplementary Information:**

The online version contains supplementary material available at 10.1186/s12874-025-02520-6.

## Clinical trial number

Not applicable.

## Background

The integration of historical knowledge into ongoing clinical studies, known as information-borrowing, allows new studies to leverage relevant patient-related information from prior research. This approach can potentially reduce the required sample size, the trial duration, and associated ethical and economic costs [1, 2], provided specific conditions are met, such as ensuring exchangeability between historical and current studies [3]. The pioneering work of Pocock et al. [4] underpins this approach. From a statistical perspective, Bayesian methods offer notable advantages, as they can integrate prior information with current data on a parameter or quantity of interest. Bayesian Dynamic Borrowing (BDB), in particular, has gained prominence as a method that dynamically adjusts the extent of historical information borrowing based on the congruence between past and current data. Its applicability has been increasingly acknowledged in numerous recent clinical studies [5–13]. Unlike static borrowing methods, the key feature of BDB is that it does not require the extent of borrowing to be predetermined by an arbitrary weight assigned to historical data—an approach strongly discouraged by regulatory authorities [2].

In recent years, various BDB methods have been developed that leverage the inclusion of elicited informative priors derived from historical data. Prior elicitation is the process of obtaining knowledge from a source to construct a prior distribution for the parameter of interest (*θ*) [14]. This prior knowledge can be derived from either expert opinion (subjective prior) or relevant empirical data (objective prior) [15, 16]. The elicited prior is then combined with the likelihood function, which represents the information contained in the observed data, to obtain the posterior distribution of the parameter. When historical data is used without discounting, a full-borrowing approach is applied. However, adopting a full-borrowing strategy is generally discouraged, as it can substantially inflate the Type I error (T1E) rate [17]. Some examples to discount the prior information were given by Ibrahim and Chen (2000) and Ibrahim et al. (2003) who introduced the power prior method [18, 19], which was later extended by Duan et al. (2006) into the modified power prior (MPP), where the discounting parameter of the power prior is specified as a distribution instead of a specific value [20]; Neuenschwandera et al. who wrote an important note about the power prior, proposing a normalized power prior (2009) and developed (2010) the meta-analytic-predictive prior (MAP) [21, 22]; Hobbs et al. (2011) who proposed the commensurate prior [23]; Schmidli et al. (2014) who modified the MAP into the robust meta-analytic-predictive prior (RMAP) that leads into a hybrid prior such that it is the weighted average of a non-informative prior and MAP [24]. More recently, Jiang et al. (2023) introduced the elastic prior [25] while Yang et al. (2023) developed the Self-Adapting Mixture (SAM) prior [26].

However, other notable studies have proposed solutions to address the practical challenge of determining how much information can be borrowed from the historical control during the design phase of a trial, as patient data from the trial are not yet available. Among these, Kotalik et al. (2022) proposed a Bayesian group-sequential trial (GSD) design based on Multisource Exchangeability Models, enabling dynamic borrowing of historical information at interim analyses. They addressed critical issues such as determining decision boundaries in a Bayesian setting, selecting the prior probability of exchangeability, and managing T1E inflation under the “local” null hypothesis, where historical data are generated under the alternative hypothesis and current trial data under the null hypothesis [27]. Zhang et al. (2022) introduced a Bayesian GSD for randomized biosimilar clinical trials, incorporating adaptive information borrowing from historical data. Their approach utilized the elastic meta-analysis predictive prior methodology to adaptively borrow information from the historical data of the reference product, facilitating go/no-go decisions at interim analyses based on the congruence between observed trial data and historical datasets [28]. More recently, Guo et al. (2024) presented an adaptive hybrid control design that combines a GSD with propensity score weighting. This method maps historical data to current trial data at each interim analysis, dynamically updating the contribution of historical control data to the current trial [29].

In the same vein, this paper aims to propose a Bayesian GSD with dynamic historical borrowing in the context of clinical trials for medical devices. Given the rapid development of medical device technology and the relatively short life cycle of these products compared to pharmaceuticals, it is crucial to assess their safety and efficacy efficiently. Bayesian methods offer a promising approach to address these needs, enabling more flexible and timely evaluations. In addition, because medical devices often evolve from earlier versions with similar mechanisms, clinical data from early versions could serve as valuable preliminary information to be borrowed for a new version of the device [30].

Specifically, our design utilizes the SAM framework [26]. In fact, the authors demonstrated how this approach possess desirable finite-sample and large-sample properties, ensuring information-borrowing consistency, and helps to circumvent selection bias and potential data dredging, which could impact the fixed-weight mixture prior approach. Moreover, the simulation study showed that SAM outperforms fixed-weight mixture priors and other existing methods such as commensurate prior and power prior, leading to a better adaptation to prior-data conflict. Moreover, the SAM prior, being analytically tractable, significantly simplifies both posterior inference and computational demands. Finally, by explicitly defining the clinically significant difference in the congruence test between current and historical data, this approach offers notable advantages in interpretability compared to other dynamic borrowing methods. It also fosters more seamless collaboration between statisticians and clinicians, ensuring that the design not only adheres to rigorous statistical standards but also increases clinical acceptability, ultimately enhancing the decision-making process in medical device trials [31].

## Methods

### Prior elicitation through Self-Adaptive mixture approach

Let ${\theta _t}$ the parameter of interest in the current test arm, ${\theta _c}$ the parameter of interest in the current control arm, ${\theta _h}$ the parameter of interest in the historical control arm, and $\Delta$ the clinically significant difference (congruent margin) such that if $|{\theta _c} - {\theta _h}| \ge \Delta$ is therefore inappropriate to borrow any information from the historical data. Therefore, the hypothesis definition to test the congruence between current and historical control data is defined as follows:

1 $$\eqalign{& {H_0}:|{\theta _c} - {\theta _h}| < \Delta \cr & {H_1}:|{\theta _c} - {\theta _h}| \ge \Delta \cr} $$

To estimate ${\theta _h}$, diverse historical study results have been pooled using the MAP approach [22] which effectively summarizes historical information on control arms through a Bayesian hierarchical model. Specifically, to derive the posterior distribution of ${\theta _h}$, we first employed the Markov Chain Monte Carlo (MCMC) method [32] and subsequently applied the Expectation-Maximization (EM) algorithm [33] to approximate the MAP prior as a parametric distribution (from now, defined as MAP-EM distribution). However, the SAM approach defines the prior distribution of ${\theta _h}$ as a mixture, consisting of an informative component (derived from the MAP-EM distribution) and a weakly informative component, incorporated in the style of the RMAP approach [24], as follows:

2 $$\eqalign{\Pr ({\theta _h}) & = w\Pr {({\theta _h})_{MAP - EM}} \cr & \quad + (1 - w)\Pr {({\theta _h})_{weak}} \cr} $$

Where *w* is the weight (discounting parameter) given to the informative component, adaptively. The weight is proportional to the Likelihood Ratio Test (LRT) odds, which compares the probability of the current control data ${D_c}$ given the null hypothesis of congruence or given the alternative hypothesis of incongruence:

$$w \propto R=\frac{R}{{1+R}}$$ such that

3 $$R=\frac{{L({D_c}|(|{\theta _c} - {\theta _h}|<\Delta ))}}{{\hbox{max} (L({D_c}|{\theta _c} \geqslant {\theta _h}+\Delta );L(D_c|{\theta _c} \leqslant {\theta _h} - \Delta ))}}$$

In this framework, the SAM prior is able to dynamically give preference, through the *w* parameter, to the informative (weakly-informative) prior component in cases of minimal (significant) evidence of prior-data conflict, thus overcoming the fixed-weight mixture priors and bias results [24].

### Effective sample size

Determining the sample size of a trial can be challenging, especially when incorporating external information via a prior distribution. The Effective Sample Size (ESS) quantifies the prior’s contribution, representing the approximate number of experimental units the prior is equivalent to. We calculated the ESS using the Expected Local Information Ratio (ELIR) method proposed by Neuenschwander et al. (2019) which has been shown to be predictively consistent—that is, the expected posterior-predictive ESS for a sample of size N equals the sum of the prior ESS and N [34]. Therefore, we have defined the ESS through the ELIR method, as follows:

4 $$ESS{\text{ }}={\text{ }}{E_\theta }{\text{ }}\left\{ {\frac{{i\left( {p\left( {{\theta _h}} \right)} \right)}}{{{i_F}({\theta _h})}}} \right\}$$

where $i\left( {p\left( {{\theta _h}} \right)} \right)= - \frac{{{\text{ }}{d^2}log{\text{ }}p\left( {{\theta _h}} \right)}}{{d{\theta _h}^{2}}}{\text{ }}$and ${i_F}\left( \theta_h \right)=1/{\theta _h}$ are the prior distribution information and the Fisher information, respectively.

### Group sequential design based on Self-Adaptive mixture approach

The BDB can improve its efficacy if nested within a GSD that allows the multiple analysis of the data during the enrolment [28]. The main idea is to update, at each interim, the *w* parameter to dynamically incorporate historical information on the control product based on the observed degree of congruence between past and newly accumulated data. This procedure can adaptively inform the go/no-go decisions by assessing Bayesian posterior probabilities of the parameters of interest in the control and test groups. The GSD + BDB algorithm, can be summarized as follows:

- Define the GSD with futility ${C_{futility,i}}$ and the efficacy boundaries ${C_{efficacy,i}}$ for the early stopping of the trial, where i = 1,…,f indicates the interim (i) and final (f) analyses (possible choices for decision boundary selection are discussed later)
- Calculate the maximum sample size N for the GSD as no historical information was borrowed, using the equal allocation proportion for randomization, such that N_t_ = N_c_ = $\:\frac{N}{2}$, where N_t_ and N_c_ are the total number allocated to the test and control groups by design, respectively.
- Let η a reasonable information fraction which allows sufficient amount of data for each interim analysis. Enroll N_*_η patients and randomize them to test or control groups, using allocation ratio 1:1.
- Calculate the posterior probability of the alternative hypothesis:

5 $$p=\Pr ({H_1}|{D_c},{D_t})$$

where ${D_c},{D_t}$ are the current control and current test data, respectively.

e. Test the alternative hypothesis:

- If $p<{C_{futility,1}}$conclude the trial early for futility;
- If $p>{C_{efficacy,1}}$, conclude the trial early for efficacy;

f. If ${C_{futility,1}} \leqslant p \leqslant {C_{efficacy,1}}$, estimate the SAM prior and *w* - using the formulas in [2] and [3], respectively - based on the current control data and calculate the prior contribution through the ESS formula in [4].
g. Enroll additional N_*_η - ESS patients so that the probability of assignment to the control arm is:

6 $$\frac{{\frac{{max({N_c}*\eta {\text{ }}-ESS,{\text{ }}0)}}{{{N_t}*\eta }}}}{{\left( {\frac{{max({N_c}*\eta {\text{ }}-ESS,{\text{ }}0)}}{{{N_t}*\eta }}} \right)+1}}$$

h. Estimate the posterior probability of the alternative hypothesis including the SAM prior $\Pr ({\theta _h})$calculated in (f).
i. Test the alternative hypothesis:

- If $p<{C_{futility,2}}$conclude the trial early for futility;
- If $p>{C_{efficacy,2}}$, conclude the trial early for efficacy;

j. If ${C_{futility,2}} \leqslant p \leqslant {C_{efficacy,2}}$, repeat steps (f) to (i) till the final analysis. If $p>{C_{efficacy,f}},$ conclude that the test product and control product are noninferior. Otherwise conclude the inferiority.

### Decision boundary selection

The proposed algorithm provides a decision at each interim analysis by leveraging futility and efficacy boundaries to facilitate early trial termination. These boundaries aim to address multiple testing issues that arise from pairing the posterior distribution with a decision rule and making repeated decisions. While identifying such boundaries is challenging in the Bayesian framework, their use is essential, as Bayesian designs also face the issue of testing multiplicity when frequentist operating characteristics are considered [35, 36]. A potential solution involves using a grid search approach to identify, through simulation, decision boundaries that effectively control the T1E rate in finite samples; alternatively, one could leverage asymptotic normality by setting Bayesian boundaries as 1− (frequentist boundaries), as discussed in previous literature [27, 35]. In this study, we derived the probability boundaries as ϕ(Zi), where ϕ(.) is the cumulative density function of the standard normal distribution, and Zi represents the frequentist boundary on the Z-scale at each interim analysis (i = 1,…,f). Particularly, we used the Hwang-Shih-DeCani spending functions with gamma = -2 for the β-spending (lower bound), and with gamma = -4 for the α-spending (upper bound).

### Power and type I error rate

The Power and T1E rate of the Bayesian design are defined as the percentages of simulated trials that lead to rejection of the null hypothesis, when the simulation is conducted under the alternative or the null hypothesis, respectively. Given the maximum sample size and the boundaries for early stopping, in cases of insufficient Power under the alternative hypothesis or excess T1E rate under the null hypothesis, we can decide to increase the sample size or modify the probability thresholds at the boundaries through a grid search to reach the nominal values of Power (1-β) and T1E rate (α).

### Motivating example

A phase 4 clinical trial on cardiovascular medical device has been used as motivating example. The primary endpoint was a composite ischemic endpoint (binary outcome). The goal of the trial was to evaluate the noninferiority of a new generation device used for the patent foramen ovale closure to the best-in-class device (“control device”). Moreover, four historical studies involving the control device have been selected for the informative prior elicitation. The hypotheses for noninferiority testing were defines as follow:

7 $$\eqalign{& {H_0}:{\theta _t} - {\theta _c} \ge M \cr & {H_1}:{\theta _t} - {\theta _c} < M \cr} $$

Where *M* is the noninferiority margin. To estimate the sample size for the trial, α = 0.05, β = 0.2, and the allocation 1:1 have been set. Moreover, 2 interims plus the final analysis have been considered for the GSD.

### Simulation study

Considering ${\theta _c}$ and ${\theta _h}$ congruent if ${\theta _h} - \Delta <{\theta _c}<{\theta _h}+\Delta$, eight scenarios have been accounted for into the simulation study (1,000 simulation). The scope was to verify the operating characteristics of the design under congruence and under increasing levels of incongruence between past and current data on the control device, as detailed in Table 1.

**Table 1:** Simulating scenarios

S	Congruence	Hypothesis	${\theta _h}$	${\theta _c}$	${\theta _t}$	$\Delta$	M
1	yes	H_0_	${\theta _h}$	${\theta _h}$	${\theta _h}$+ m	δ	m
2	yes	H_1_	${\theta _h}$	${\theta _h}$	${\theta _h}$	δ	m
3	no	H_0_	${\theta _h}$	${\theta _h}$- δ	(${\theta _h}$- δ) + m	δ	m
4	no	H_1_	${\theta _h}$	${\theta _h}$- δ	${\theta _h}$- δ	δ	m
5	no	H_0_	${\theta _h}$	${\theta _h}$- δ*3/2	(${\theta _h}$- δ*3/2) + m	δ	m
6	no	H_1_	${\theta _h}$	${\theta _h}$- δ*3/2	${\theta _h}$- δ*3/2	δ	m
7	no	H_0_	${\theta _h}$	${\theta _h}$- δ*2	(${\theta _h}$- δ*2) + m	δ	m
8	no	H_1_	${\theta _h}$	${\theta _h}$- δ*2	${\theta _h}$- δ*2	δ	m

S: Scenario; ${\theta _h}$: estimated proportion of events in the historical controls; ${\theta _c}$: Expected proportion of events in the current controls; ${\theta _t}$: Expected proportion of events in test arm; $\Delta$: clinically significant difference between historical and current control parameters; M: the noninferiority margin

Moreover, for each scenario, the version (a) and (b) correspond to results from (a) the proposed GSD + BDB and (b) the GSD with fixed prior (with no update at the interim) defined under congruence ${\theta _c}={\theta _h}$.

## Results

**Fig. 1:**
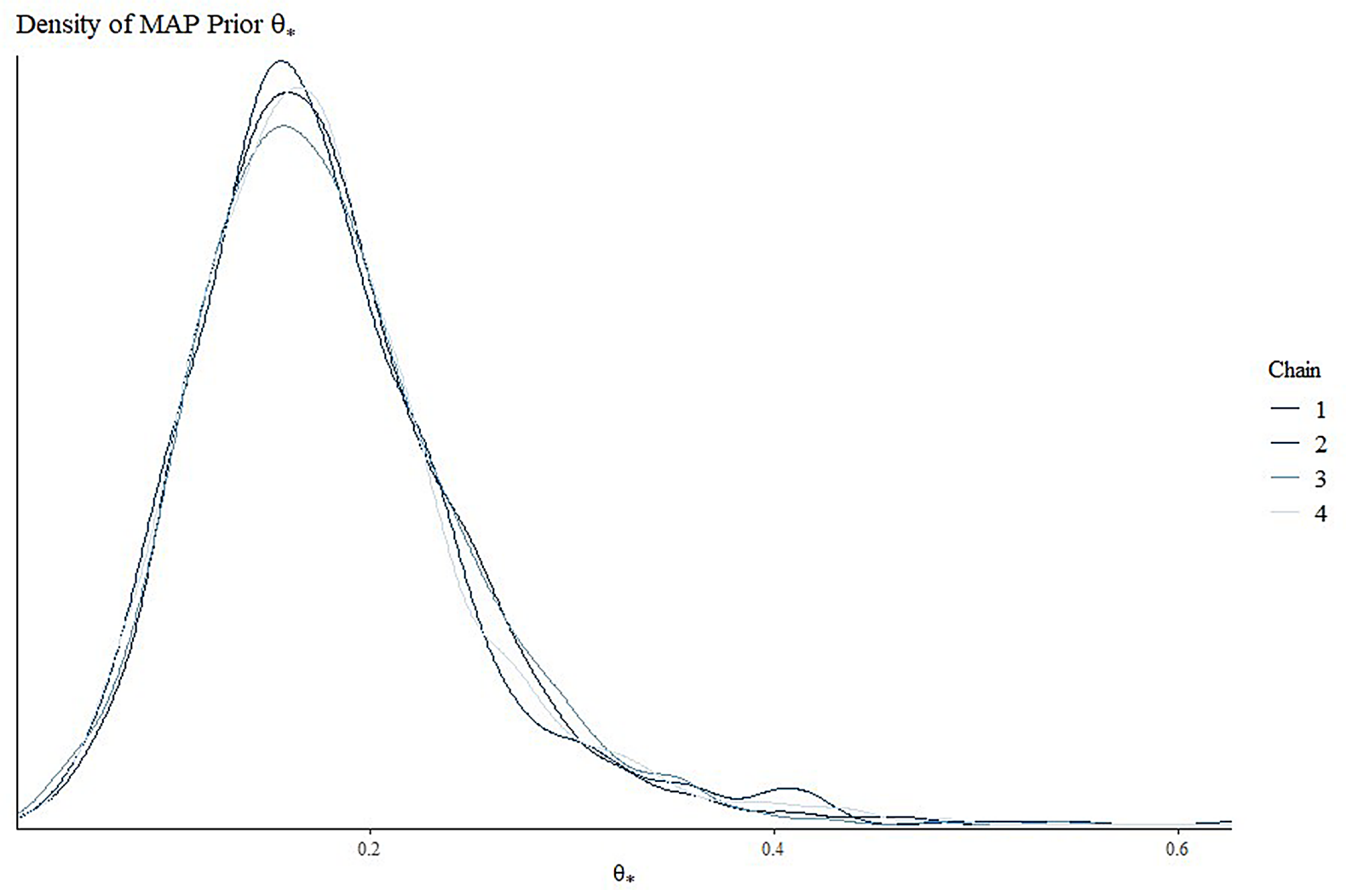
Density of MAP prior for θ_h_ by posterior MCMC samples

**Fig. 2:**
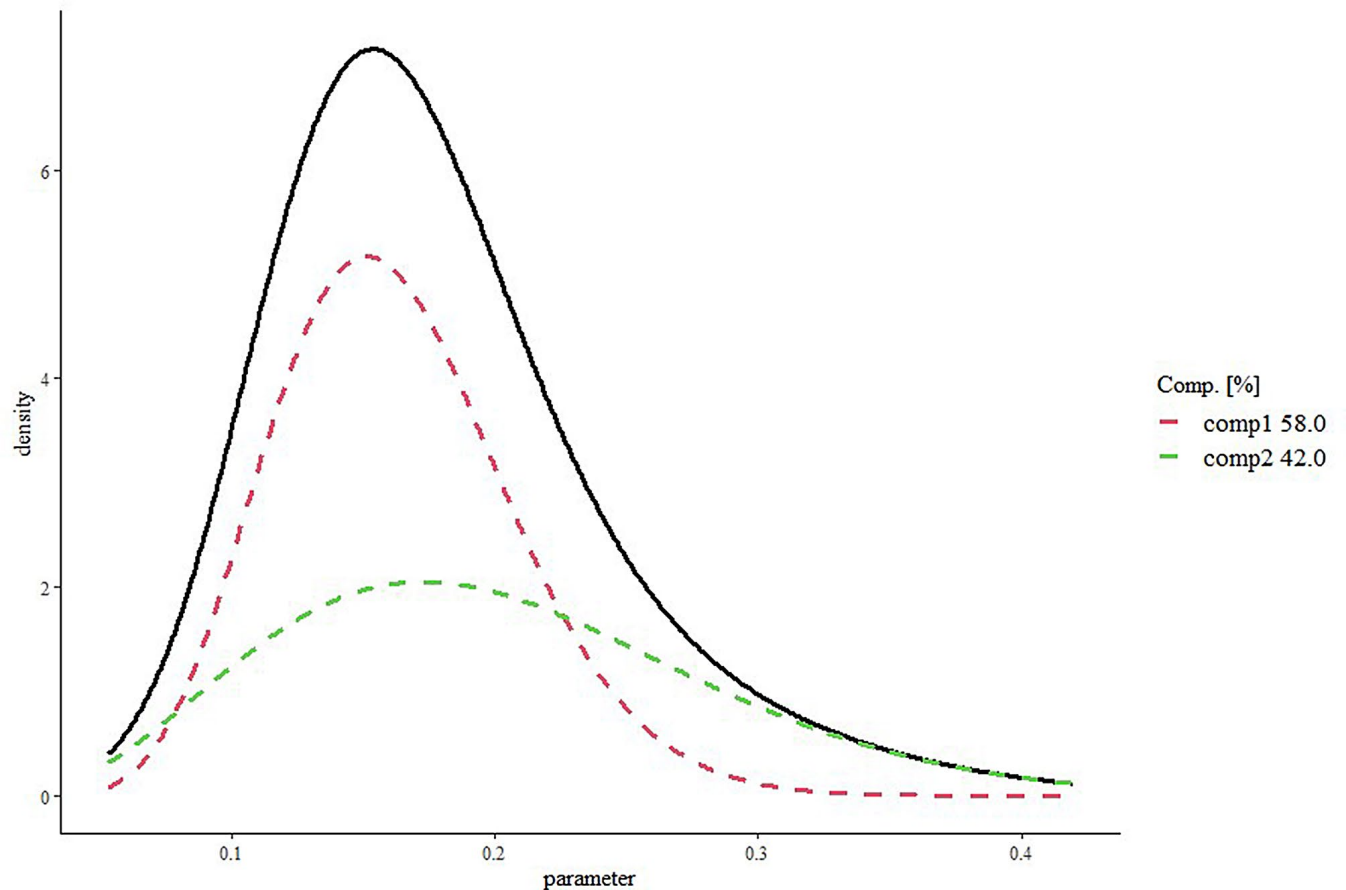
Density of MAP prior approximated by EM algorithm as mixture of beta densities

The four historical studies on the control device included 204, 220, 499, and 60 patients, with 30, 20, 14, and 10 events, respectively. The heterogeneity between studies - estimated through the statistics ${I^2}$- was equal to 92% (*p* < 0.001). Figure 1 illustrates the MAP prior for ${\theta _h}$ derived from the posterior MCMC samples (four chains), based on the historical data. Additionally, Fig. 2 depicts the density of MAP prior approximated by the EM algorithm, as a mixture of beta densities. In this case, the MCMC sample is well approximated by a mixture of two components distributed as $Beta(\alpha =10.59,\beta =54.71)$ and $Beta(\alpha =4.53,\beta =17.92)$ with weights equal to 0.58 and 0.42, respectively. The average of the distribution ${\widehat {\theta }_h}$ was 0.179 (95% credible interval: 0.08–0.33). Therefore, since under congruent scenario ${\widehat {\theta }_h}={\widehat {\theta }_c}$= 0.179, the noninferiority margin (m) was set equal to 0.04 and the significant clinical difference (δ) were fixed equal to 0.02.

**Fig. 3:**
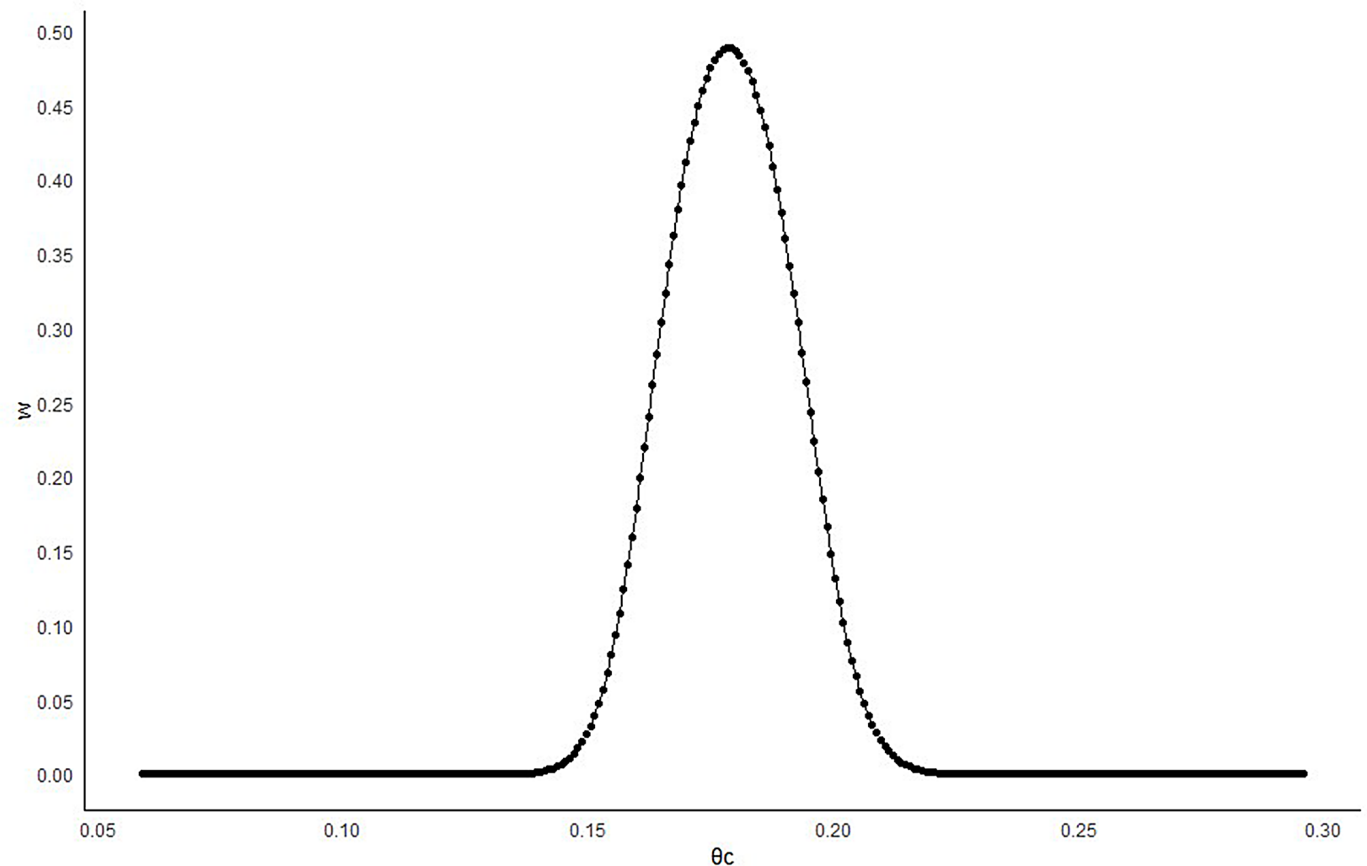
Estimated MAP weight (w) according to the incidence in the current control arm

Figure 3 shows the estimated average of the *w* parameter based on the incidence of the primary endpoint in the current control arm. The mean reaches 50% when ${\widehat {\theta }_c}={\widehat {\theta }_h}$, while it decreases rapidly when ${\hat\theta _c} \ge {\hat\theta _h} + 0.02\,{\rm{or}}\,{\hat \theta _c} \le {\hat \theta _h} - 0.02$  

The sample size required to reach at least 80% of Power and to control the T1E rate under 5%, using a frequentist one-stage design, was 2,282. After the adjustment for the GSD, the maximum sample size required was 2,386. The Z-values corresponding to the frequentist lower bounds were − 0.42, 0.64, 1.64, while those corresponding to the frequentist upper bounds were 2.79, 2.29, and 1.64. Therefore, in Table 2 we describe the Bayesian decision regions, based on the posterior probability of H_1_.

**Table 2:** Decision boundaries applied on the posterior probability of H_1_ for the early stopping in the Bayesian group sequential design

Analysis	Futility Stopping	Continuation Region	Efficacy Stopping
1	Pr(H_1_) < 0.337	0.337 ≤ Pr(H_1_) ≤ 0.997	Pr(H_1_) > 0.997
2	Pr(H_1_) < 0.739	0.739 ≤ Pr(H_1_) ≤ 0.989	Pr(H_1_) > 0.989
f	-	-	Pr(H_1_) > 0.949

Table 3 reports the results across simulated studies for design version a). Specifically, it shows the average Power and T1E rate, the expected number of enrolled patients, the expected ESS, the percentage of studies early stopped and the estimated *w* at each interim analysis.

**Table 3:** Results across 1,000 simulated studies for design version a). Average values and interquartile ranges provided

S	T1E/ Power	Enrolled patients	ESS	%Stop (1st interim)	%Stop (2nd interim)	*w* (1st interim)	*w* (2nd interim)
1	0.044*	1497 (796–1586)	16 (10–18)	33	44	0.47 (0.4–0.56)	0.42 (0.25–0.59)
2	0.8	1850 (1576–2358)	20 (12–28)	13	38	0.45 (0.37–0.56)	0.52 (0.40–0.66)
3	0.048*	1506 (796–1778)	15 (8–19)	34	41	0.4 (0.28–0.52)	0.47 (0.33–0.62)
4	0.83	1835 (1578–2376)	10 (5–15)	15	39	0.36 (0.24–0.49)	0.21 (0.08–0.3)
5	0.05*	1509 (796–2350)	11 (4–15)	35	39	0.32 (0.20–0.44)	0.29 (0.12–0.44)
6	0.85	1829 (1582–2381)	6 (2–9)	14	41	0.29 (0.16–0.41)	0.09 (0.03–0.12)
7	0.04*	1480 (796–1590)	6 (2–7)	35	44	0.20 (0.11–0.26)	0.13 (0.05–0.17)
8	0.88	1792 (1586–2383)	3 (1–4)	15	44	0.19 (0.1–0.25)	0.03 (0.01–0.04)

T1E: Type I Error rate (*); ESS: Effective Sample Size

Table 4 presents the same results as Table 3 but considering the design versions b). Moreover, Table S1 shows the results considering a GSD with 4 rather than 3 analyses for design version a).

**Table 4:** Results across 1,000 simulated studies for design version b). Average values and interquartile ranges provided

S	T1E/ Power	Enrolled patients	ESS	%Stop (1st interim)	%Stop (2nd interim)	*w* (1st interim)	*w* (2nd interim)
1	0.052*	1511 (796–1578)	17 (13–26)	32	44	0.5 (0.5–0.5)	0.5 (0.5–0.5)
2	0.8	1846 (1578–2360)	20 (13–26)	13	41	0.5 (0.5–0.5)	0.5 (0.5–0.5)
3	0.052*	1524 (796–2360)	17 (13–26)	34	38	0.5 (0.5–0.5)	0.5 (0.5–0.5)
4	0.83	1806 (1578–2360)	20 (13–26)	14	42	0.5 (0.5–0.5)	0.5 (0.5–0.5)
5	0.061*	1490 (796–1578)	18 (13–26)	35	41	0.5 (0.5–0.5)	0.5 (0.5–0.5)
6	0.87	1821 (1578–2360)	20 (13–26)	12	44	0.5 (0.5–0.5)	0.5 (0.5–0.5)
7	0.055*	1512 (796–2360)	18 (13–26)	34	41	0.5 (0.5–0.5)	0.5 (0.5–0.5)
8	0.87	1784 (1578–2360)	19 (13–26)	15	44	0.5 (0.5–0.5)	0.5 (0.5–0.5)

T1E: Type I Error rate (*); ESS: Effective Sample Size

## Discussion

This paper introduces a Bayesian GSD incorporating dynamic historical borrowing, tailored for clinical trials involving medical devices. Particularly, the proposed methodology leverages the SAM prior framework to adaptively integrate historical data. A motivating example from a medical device trial is presented to demonstrate the practical implementation of this approach, offering valuable insights into the design’s operating characteristics under both congruent and incongruent scenarios.

### Main results

The proposed design, when evaluated under scenarios where historical and current data were congruent (scenarios 1 and 2), demonstrated a significant reduction in the number of enrolled patients (1,497 vs. 2,386 under H_0_​ and 1,850 vs. 2,386 under H_1_​) while maintaining both the T1E and Power at their nominal levels. Additionally, the asymmetric early stopping boundaries enabled a higher proportion of trials to terminate early under the null hypothesis (33% at the first interim and 44% at the second interim), supporting an ethical trial design [37].

In scenarios involving incongruence (scenarios 3 to 8), the weight estimated through the SAM method and the ESS both decreased. Notably, in the example analysed, the ESS was relatively small even under congruence. This outcome can be attributed to the high variance of the parameter ${\theta _h}$ estimated through the MAP prior. Moreover, the ELIR method effectively captured the precision of the historical parameter estimates, such that lower precision led to smaller ESS values. This characteristic is desirable as it accounts for the heterogeneity underlying the historical studies used for prior elicitation. In this case, the heterogeneity was substantial, with ${I^2}$ estimated at 92%, reflecting significant variation in the event proportions across the four historical studies in the control arm. Consequently, the conservatively small ESS mitigated potential bias.

When design version (b) was considered, the weight was fixed at 0.5, corresponding to the average weight assigned to the informative prior component by the SAM method under the proposed congruent scenarios (see Fig. 3). This approach led to an increase in T1E rate and prevented the ESS from decreasing under incongruence. While the observed T1E rate increase was not substantial in this example, a highly precise estimate of ${\theta _h}$ could significantly exacerbate the bias in the presence of prior data conflict, potentially leading to detrimental impacts on the trial results.

### Design advantages and limitations

The proposed design offers several key advantages. First, it enhances resource efficiency by dynamically integrating historical data, which reduces the need for enrolling new patients. This is particularly beneficial in trials with limited budgets or rare events, where patient enrollment can be time-consuming and expensive [38]. Second, the design allows for early stopping for efficacy or futility. By incorporating sequential analyses, trials can conclude once sufficient evidence is collected, avoiding the need to unnecessarily expose patients to investigational treatments. This ensures ethical responsibility by minimizing patient risk, which is increasingly important in medical device trials where safety is a paramount concern [39]. Moreover, the adaptive weighting of historical information ensures that bias is minimized [26]. If the new data conflicts with historical data, the algorithm discounts the historical data appropriately, maintaining control over the T1E rate. This dynamic borrowing mechanism thus ensures robustness against incongruent scenarios, preserving the trial’s integrity even when historical data differ from current observations. Another advantage is the flexibility offered by this design. Parameters such as the congruence threshold (δ), the number of interim analyses, and the spending functions used to derive Bayesian stopping boundaries can be customized to address specific clinical and statistical requirements. This flexibility enables a tailored approach, ensuring the design is well-suited to the unique context and objectives of each study. Finally, the design complies with regulatory demands, such as those imposed by the European Union’s Medical Device Regulation (MDR), which requires stringent evidence for device approval [40]. The proposed method facilitates quicker, yet rigorous evaluations, supporting faster innovation in the medical device field while maintaining high standards for safety and efficacy.

Although the BDB is increasingly used in the literature in conjunction with covariate adjustment techniques such as inverse treatment probability weighting or G-computation [41], the present study only uses aggregated historical estimates with respect to the parameter of interest. However, if individual-level historical data were available, the design could be further refined by selecting historical samples with baseline and medical characteristics more closely aligned with those of the current sample. Additionally, this study focuses solely on the case of binary outcomes. While this is appropriate for many clinical endpoints, the proposed method could be easily extended to handle continuous or count outcomes. Future research will explore these extensions.

## Conclusions

In summary, this paper highlights the potential benefits of integrating BDB with GSD in the rapidly advancing field of medical device technology. The SAM prior was employed as a dynamic mechanism to appropriately discount historical data at both the design and interim analysis stages, ensuring that the trial remains robust and responsive to the data.

Notably, the SAM prior offers significant interpretability advantages facilitating effective collaboration between statisticians and clinicians to ensure the design meets both statistical rigor and clinical relevance. By enabling the reduction of control sample sizes through the reliable borrowing of information from prior studies and incorporating ethical considerations via early stopping boundaries, the proposed approach establishes itself as a powerful and adaptable tool for advanced clinical trial design.

## Electronic supplementary material

Below is the link to the electronic supplementary material.

Supplementary Material 1

## Data Availability

No datasets were generated or analysed during the current study.

## Abbreviations

BDB: Bayesian Dynamic Borrowing
ESS: Effective Sample Size
ELIR: Expected Local Information Ratio
EM: Expectation Maximization
GSD: Group Sequential Design
LRT: Likelihood ratio test
MAP: Meta-Analytic-Predictive
MCMC: Markov Chain Monte Carlo
SAM: Self-Adapting Mixture

## Symbols

D_h_: Historical control data
D_c_: Current control data
D_t_: Current test data
θ: Parameter of interest (general)
θ_c_: Parameter of interest in the current group
θ_h_: Parameter of interest in the historical group
θ_t_: Parameter of interest in the test group
△: Congruence clinical margin
R: Likelihood ratio test / Posterior probability ratio
w: Informative prior weight
H_0_: Null hypothesis
H_1_: Alternative hypothesis
Pr(.): Probability
*p*: Posterior probability of the alternative hypothesis
η: Information fraction
M: noninferiority margin
N: Total number of patients
N_t_: Total number of patients allocated to the test group (N/2)
N_c_: Total number of patients allocated to the control group (N/2)
*C*_*futility*_: Futility boundary
*C*_*efficacy*_: Efficacy boundary
T1E: Type I Error rate
α: Significance level
β: Type II Error rate
