# Supplementary material for: Bayesian dynamic borrowing in group-sequential design for medical device studies
Source: BMC Med Res Methodol. 2025 Mar 20;25:78. doi: 10.1186/s12874-025-02520-6 (PMC11924708; doi:10.1186/s12874-025-02520-6)
Supplement: Supplementary file 1 — Supplementary Material 1 [file 12874_2025_2520_MOESM1_ESM.docx]

Here, we present design version a) for the motivating example, incorporating four analyses instead of three. The maximum sample size required, adjusted for the Group-Sequential Design with four analyses, was 2,412. The Bayesian decision boundaries for futility stopping were set at 0.221, 0.540, and 0.808, while those for efficacy stopping were 0.998, 0.995, 0.985, and 0.949.

Table S1: Results across 1,000 simulated studies for design version a) including 4 analyses. Average values and interquartile ranges provided.

| S | T1E/ Power | Enrolled  patients | ESS | %Stop  (1^st^ interim) | %Stop  (2^nd^ interim) | %Stop  (3^rd^ interim) | $w$  (1^st^ interim) | $w$  (2^nd^ interim) | $w$  (3^rd^ interim) |
| --- | --- | --- | --- | --- | --- | --- | --- | --- | --- |
| 1 | 0.047* | 1409  (1190-1787) | 20 (11-27) | 21 | 37 | 27 | 0.45  (0.38-0.54) | 0.48  (0.36-0.61) | 0.41  (0.24-0.58) |
| 2 | 0.79 | 1789  (1201-2365) | 26 (15-36) | 6 | 22 | 37 | 0.43  (0.36-0.53) | 0.49  (0.40-0.61) | 0.53  (0.40-0.69) |
| 3 | 0.045* | 1409  (1190-1791) | 18 (9-26) | 22 | 36 | 27 | 0.38  (0.29-0.49) | 0.41  (0.28-0.56) | 0.47  (0.30-0.64) |
| 4 | 0.85 | 1763  (1199-2386) | 15 (8-21) | 7 | 23 | 38 | 0.43  (0.34-0.54) | 0.33  (0.17-0.46) | 0.20  (0.07-0.27) |
| 5 | 0.048* | 1407  (1191-1799) | 13 (6-18) | 24 | 35 | 25 | 0.31  (0.21-0.41) | 0.29  (0.15-0.40) | 0.28  (0.11-0.40) |
| 6 | 0.855 | 1768  (1201-2399) | 9 (4-13) | 8 | 24 | 36 | 0.35  (0.23-0.46) | 0.17  (0.07-0.24) | 0.06  (0.02-0.08) |
| 7 | 0.051* | 1400  (1190-1804) | 8 (3-11) | 25 | 32 | 29 | 0.23  (0.14-0.30) | 0.15  (0.06-0.20) | 0.11  (0.03-0.15) |
| 8 | 0.864 | 1744  (1202-2404) | 6 (2-8) | 7 | 27 | 35 | 0.27  (0.17-0.35) | 0.10  (0.04-0.13) | 0.022  (0.005-0.025) |

T1E: Type I Error (*); ESS: Effective Sample Size
